# Lentivirus enables the detection of strand-specific ssDNA gaps by the DNA fiber spreading assay

**DOI:** 10.1038/s42003-026-10367-w

**Published:** 2026-05-26

**Authors:** Melisande Wong, Xupei Ou, Evan Brown Ton, Yanjun Pan, Jieya Shao

**Affiliations:** 1 Division of Oncology, Department of Medicine, Washington University in St. Louis, St. Louis, MO USA; 2 Siteman Cancer Center, Washington University in St. Louis, St. Louis, MO USA

**Keywords:** DNA replication, Biological techniques

## Abstract

The single-molecule DNA fiber spreading assay is widely used to study DNA replication fork dynamics. However, tight sister chromatid cohesion during DNA spreading causes the leading and lagging strands to collapse into a single fiber, masking strand-specific alterations that are increasingly recognized as biologically and therapeutically important. Here, we report an unexpected finding that lentiviral infection of human cell lines prior to DNA spreading enables visualization of strand-specific single-stranded DNA gaps arising from defects in Okazaki fragment maturation or PrimPol overactivation. Our results suggest that this effect is transient and independent of viral vectors, encoded sequences, or genome integration. Mechanistically, lentivirus transiently reduces cohesin level on nascent DNA, indicating that loosening of sister chromatid cohesion during spreading allows daughter strand separation. We propose that lentiviral pre-exposure can be a simple, effective modification to enable the DNA fiber spreading assay to detect strand-specific DNA replication changes and overcome its limitation.

## Abstract (web summary)

Lentiviral infection transiently reduces cohesin level on nascent DNA in human cells and enables the DNA fiber spreading assay to detect strand-specific, single-stranded post-replicative DNA gaps.

## Introduction

As an essential biological process, DNA replication is tightly regulated in cells to ensure the faithful duplication of genetic material. This becomes especially important under stress conditions, when the normal progression of DNA replication forks faces disruption and adaptive changes in fork dynamics are implemented to balance genome integrity and cell survival. A central approach to experimentally studying replication fork dynamics is the DNA fiber assay, which enables genome-wide analysis of newly synthesized (nascent) DNA at single-molecule resolution. In this assay, nascent DNA is sequentially labeled with two different thymidine analogs, such as 5-iodo-2’-deoxyuridine (IdU) and 5-chloro-2’-deoxyuridine (CldU), and fork dynamics can be altered by incorporating different drug treatments before, during, or after the pulse labeling. Each thymidine analog is subsequently stained using a specific antibody, and the tract length of distinct types of DNA fibers and their relative percentages can be analyzed to reveal quantitative changes in replication parameters and fork behaviors^1–5^.

There are two main methods of depositing DNA fibers on glass for immunodetection - DNA spreading versus DNA combing. For DNA spreading, cells are spotted and lysed on the slides, which are then tilted at a 25–40 degree angle to gravitationally spread the released DNA fibers^2^. For DNA combing, cells are embedded in agarose plugs, lysed in the presence of proteinase K, and subsequently straightened onto silanized coverslips in a combing machine based on receding meniscus^3^. While DNA combing generates uniformly aligned fibers and enables accurate measurement of the tract lengths, the time and effort required for sample preparation and the need for the special combing machine collectively hinder its widespread use. In comparison, the ease, speed, and higher throughput of the DNA spreading assay make it the preferred method for studying DNA replication. Nevertheless, a major limitation of DNA spreading, as opposed to the DNA combing, is that it cannot separate the adjacent sister chromatids held together by cohesion. As a result, the leading and lagging nascent DNA strands are held together and visualized as one single fiber, thereby limiting the utility of the DNA spreading assay to detect strand-specific changes in DNA replication^6^.

In recent years, post-replicative single-stranded DNA (ssDNA) gaps have emerged as an important source of genome instability, which influence the efficacy of genotoxic cancer therapies^7,8^. These gaps, though not directly detectable by the DNA fiber assay, can be revealed indirectly by employing the ssDNA-specific endonuclease S1, which cleaves the template DNA strand at gap sites, leading to the shortening of the corresponding nascent DNA fibers^9,10^. Despite growing evidence that gaps can form on either the leading or lagging strand of the nascent DNA through distinct biological mechanisms, our ability to robustly investigate them is limited by the inability of the DNA spreading technique to resolve sister chromatids and detect strand-specific DNA replication changes. In this paper, we report a previously unrecognized ability of lentiviral infection to enable the detection of strand-specific ssDNA gaps by the DNA spreading assay, likely due to a reduction of cohesin level on nascent DNA. We suggest that lentiviral exposure of cells prior to the DNA spreading assay can be a simple modification to make it applicable to the detection of strand-specific DNA gaps.

## Results

### Single-stranded DNA gaps due to FEN1 loss-of-function by lentiviral shRNA infection, but not chemical inhibition, can be detected by DNA fiber spreading assay

**Fig. 1:**
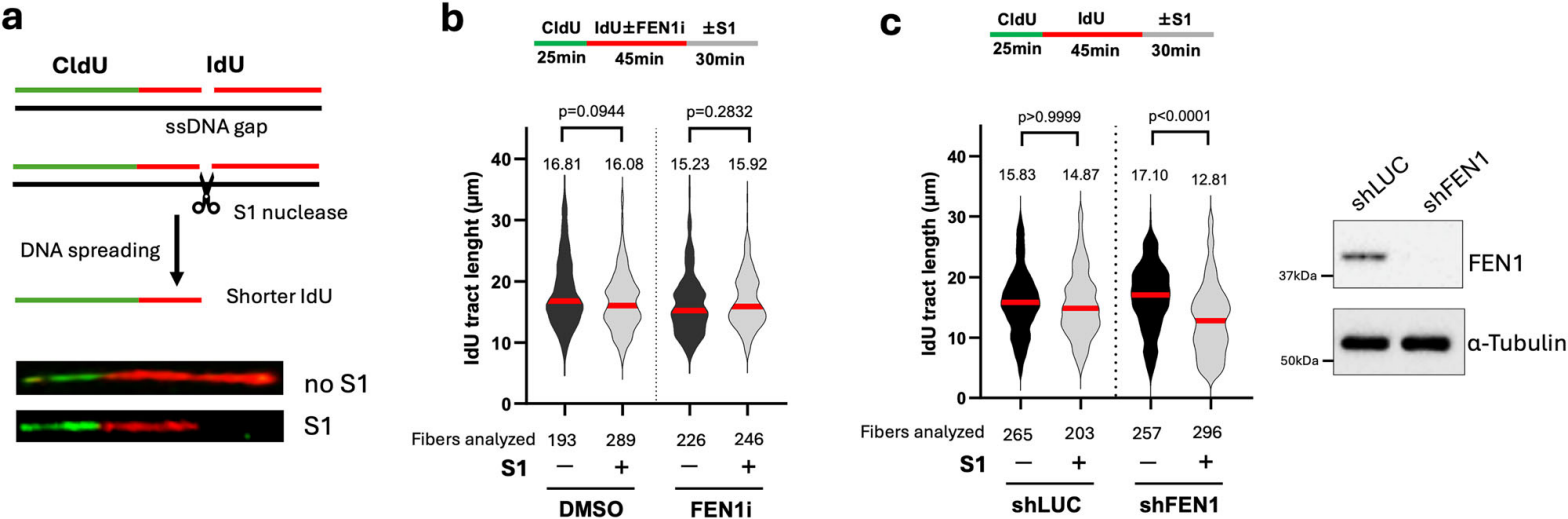
Single-stranded DNA gaps due to FEN1 loss-of-function by lentiviral shRNA infection but not chemical inhibition, can be detected by DNA fiber spreading assay. **a** Schematic of the S1 DNA fiber assay used to detect single-stranded gaps in newly synthesized DNA. **b** U2OS cells were sequentially labeled with 30 μM CldU for 25 min and 250 μM IdU for 45 min during which time 10 μM FEN1 inhibitor LNT1 was present or absent. Cells were subsequently subjected to the S1 DNA fiber spreading analysis. **c** U2OS cells were infected with lentiviral particles encoding shRNA sequences targeting firefly luciferase (shLUC) or human FEN1 (shFEN1), and subjected to the S1 DNA fiber spreading assay after four days. Western blots for FEN1 and tubulin are shown. Median IdU tract lengths are shown above each experimental group and indicated by the red bars. The exact number of dual-colored DNA fibers analyzed is shown below each experimental group. Three independent experiments were performed, which showed similar trends. *P* values are based on Kruskal–Wallis test with Dunnett’s multiple comparisons.

It was recently reported by Meroni et al. that lagging-strand-specific DNA breaks caused by the chemical inhibitor of FEN1 (LNT1) on nascent DNA in RPE-1 cells cannot be detected by the standard S1 DNA spreading assay^6^. FEN1 is a well-known Okazaki fragment processing (OFP) enzyme required for Okazaki fragment ligation and maturation, and its inactivation has been previously shown to generate ssDNA gaps^11^. In this assay, nascent DNA was first labeled with CldU alone, followed by IdU in the presence or absence of the FEN1 inhibitor. Cells were then briefly detergent-extracted to remove cytosols and treated with the ssDNA-specific S1 nuclease. Shortening of the IdU tract lengths of dual-color fibers (indicating active forks) relative to their no S1 controls would indicate the presence of gaps (Fig. 1a). Interestingly, it was shown that DNA combing, which utilizes proteinase K in the experimental procedure, can detect FEN1 inhibitor-induced gaps, consistent with the idea that cohesion is disrupted to separate the sister chromatids^6^. Indeed, using FEN1 inhibitor-treated U2OS cells, we did not detect any gaps by the S1 DNA spreading assay (Fig. 1b). Unexpectedly, however, when FEN1 was silenced in U2OS cells via lentiviral infection of shRNAs four days prior to DNA spreading, the gaps were readily detectable (Fig. 1c). Notably, the fiber tract length without S1 cleavage appeared longer in the FEN1 knockdown cells compared to the control cells (Fig. 1c), consistent with a prior report of a fork-speeding phenotype associated with impaired Okazaki fragment maturation^12^. This unexpected difference suggests that genetic loss-of-function of FEN1 by lentivirus-based shRNA knockdown somehow enables the DNA spreading assay to detect ssDNA gaps specifically on the lagging strand.

### Lentiviral exposure enables DNA spreading assay to detect ssDNA gaps caused by FEN1 inhibition or PrimPol overexpression

**Fig. 2:**
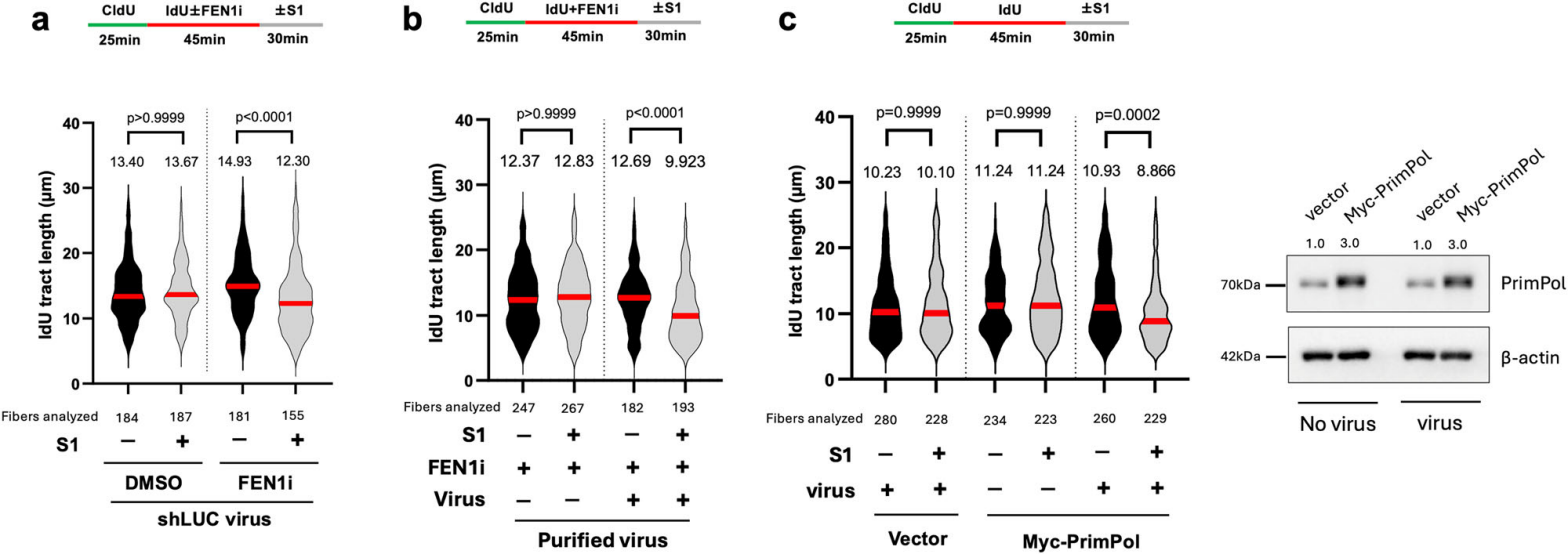
Lentiviral exposure enables the detection of strand-specific ssDNA gaps caused by FEN1 inhibitor or PrimPol overexpression by the S1 DNA spreading assay. **a** U2OS cells were infected with lentiviral particles encoding shLUC in the pLKO.1 vector. On day 4 post-infection, cells were subjected to the S1 DNA spreading assay with and without FEN1 inhibitor and analyzed as in Fig. 1. **b** U2OS cells were infected or not with 0.5 μL of purified shLUC lentivirus (MOI of 5) diluted by 1 ml fresh medium. On day 7 post-infection, cells were subjected to the S1 DNA fiber spreading assay with FEN1 inhibitor and analyzed as in Fig. 1. **c** Control or Myc-PrimPol-overexpressing U2OS cells were infected or not with shLUC lentivirus, and subjected on day 4 post-infection to the S1 DNA fiber spreading assay. Western blot was performed using lysates from uninfected or shLUC-infected control and Myc-PrimPol-overexpressing cells 4 days post-infection, and the relative expression of PrimPol (endogenous plus Myc-PrimPol) was quantified by densitometry. For DNA fiber data, median IdU tract lengths are shown above each experimental group and indicated by the red bars. The exact number of dual-colored DNA fibers analyzed is shown below each experimental condition. Three independent experiments were performed, which showed similar trends. *P* values are based on Kruskal–Wallis test with Dunnett’s multiple comparisons.

We next asked if it is the lentiviral exposure itself that renders strand-specific gap detection by the DNA spreading assay. If true, exposure of cells to lentivirus encoding a non-targeting shRNA should enable the DNA spreading assay to detect gaps caused by chemical inhibition of FEN1. To test this, we treated U2OS cells with lentiviral particles encoding a control shRNA targeting firefly luciferase four days prior to the S1 DNA fiber spreading assay with and without FEN1 inhibitor. We found that lentiviral infection alone without FEN1 inhibition did not produce any gaps (DMSO group, Fig. 2a). However, shLUC lentivirus together with FEN1 inhibitor produced gaps that are readily detectable by the DNA spreading assay. To confirm this finding, we performed the same experiment using lentiviral particles encoding the yellow fluorescence protein (YFP) expressed from a different transfer vector. We observed the same result as in shLUC-infected cells (Supplementary Fig. 1a). Thus, our results suggest that lentiviral exposure per se, rather than the expression of specific encoded transgenes, enables the DNA spreading assay to detect strand-specific gaps.

Given that the lentiviral particles used in our experiments are in the crude form of conditioned media directly collected from transfected HEK293T cells, we next asked if it is the virus or other secreted components present in the conditioned media that triggered the effect on the DNA spreading assay. As such, we transfected HEK293T cells with shLUC transfer vector with and without the necessary packaging plasmids to produce virus, collected the conditioned media in parallel, and exposed U2OS cells to them five days prior to the S1 DNA spreading assay with and without FEN1 inhibitor. We observed that, while cells exposed to the virus-containing medium showed detectable ssDNA gaps, those exposed to the virus-free conditioned medium showed none (Supplementary Fig. 1b). To further confirm that the effects are virus-driven, we performed the orthogonal experiment where we infected U2OS cells with a small volume (0.5 μL) of purified shLUC lentivirus diluted 2000-fold in fresh growth medium. Based on the viral titer, the multiplicity of infection (MOI) needed for complete resistance to puromycin (selection marker on the viral vector) was around 5. Indeed, cells pre-infected with the purified virus showed FEN1 inhibitor-induced ssDNA gaps detectable by the DNA spreading assay while the uninfected cells showed none (Fig. 2b). We found no evidence of increased sensitivity of virally infected U2OS cells to FEN1 inhibition compared to uninfected control cells based on the similar degrees of growth suppression by 10 μM FEN1 inhibitor in these cells (Supplementary Fig. 1c). This indicates similar degrees of OFP interference by FEN1 inhibitor and consequent lagging-strand gap induction in the control and virally infected cells. We also did not detect any evidence of virus-induced DNA replication stress four days post-infection, based on the low baseline levels of pSer^784^-VCP and pSer^4/8^-RPA32 as compared to those induced by 2 mM HU treatment (positive control)^13,14^ (Supplementary Fig. 1d). Collectively, these data suggest that lentivirus enables the DNA spreading to detect strand-specific DNA gaps.

To further test this hypothesis, we sought to induce strand-specific ssDNA gaps using an alternative approach. PrimPol, a recently identified DNA-directed primase and polymerase, has emerged as a key player in the adaptive response of replication forks to stress-induced stalling^15,16^. By repriming DNA synthesis downstream of replication-blocking lesions, PrimPol enables replication forks to continue progressing while inevitably leaving behind ssDNA gaps that are subsequently filled in post-replicatively^17–21^. It is generally accepted that PrimPol primarily acts on the leading strand, as the inherently discontinuous Okazaki fragments on the lagging strand are primed by the dedicated Polα^22,23^. Because genotoxic treatments likely interfere with the synthesis of both daughter strands indiscriminately and generate gaps on each through distinct mechanisms, we relied on forced PrimPol upregulation in otherwise unperturbed cells to drive spontaneous repriming events preferentially on the leading strand. Indeed, overexpression of Myc-tagged PrimPol in U2OS cells at approximately threefold above the endogenous level resulted in the formation of nascent ssDNA gaps that were detectable by the S1 DNA fiber spreading assay only following prior infection using the control shLUC lentivirus (Fig. 2c). In contrast, neither virally infected control cells nor cells overexpressing Myc-PrimPol without prior viral infection showed detectable DNA gaps in this assay (Fig. 2c). These results suggest that PrimPol overexpression induces strand-specific nascent DNA gaps in otherwise unperturbed U2OS cells and further support the hypothesis that lentiviral infection enables the DNA fiber spreading assay to detect strand-specific DNA gaps.

### Lentivirus-induced ability of the DNA spreading assay to detect strand-specific gaps is cell line-independent

**Fig. 3:**
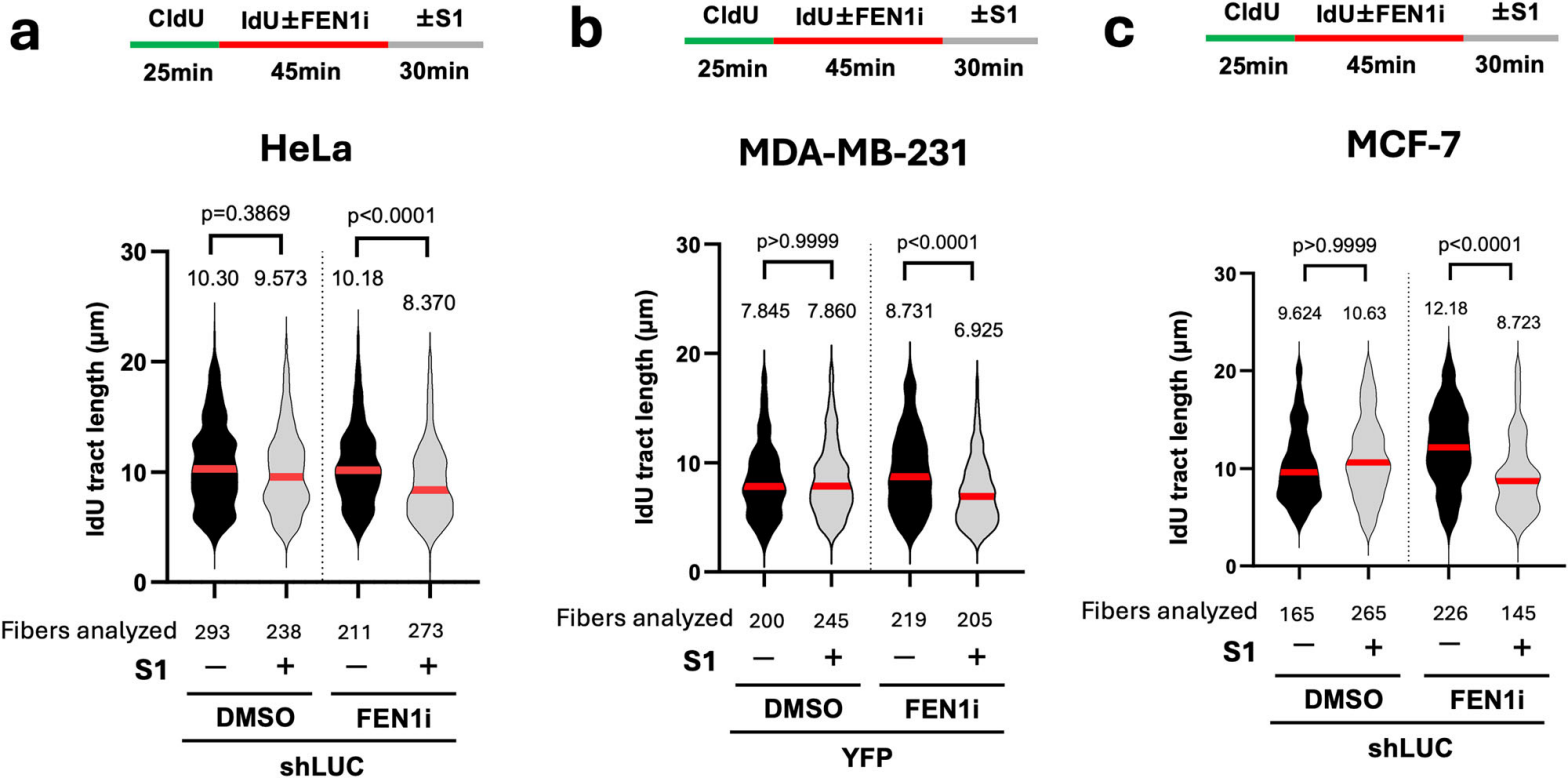
Lentivirus enables the detection of strand-specific ssDNA gaps by the S1 DNA spreading assay in different cell lines. HeLa (**a**), MDA-MB-231 (**b**), and MCF-7 (**c**) cells were infected with either shLUC or YFP-encoding lentiviral particles four days prior to the S1 DNA spreading assay with and without FEN1 inhibitor. Experimental design and data quantification were performed as described in earlier figures. Median IdU tract lengths are shown above each experimental group and indicated by the red bars. The exact number of dual-colored DNA fibers analyzed is shown below each experimental condition. Three independent experiments were performed, which showed similar trends. *P* values are based on Kruskal–Wallis test with Dunnett’s multiple comparisons.

To confirm that our finding is not cell line-specific, we infected three other human cell lines (HeLa, MDA-MB-231, and MCF-7) with lentiviral particles expressing shLUC or YFP and performed the same S1 DNA fiber assay four days later to detect FEN1 inhibitor-induced gaps on the lagging strand. Similar to U2OS cells, prior viral exposure of all three cell lines produced DNA gaps detectable by DNA spreading in a FEN1 inhibitor-dependent manner (Fig. 3a–c).

### Lentivirus-induced ability of the DNA spreading assay to detect strand-specific gaps is transient

**Fig. 4:**
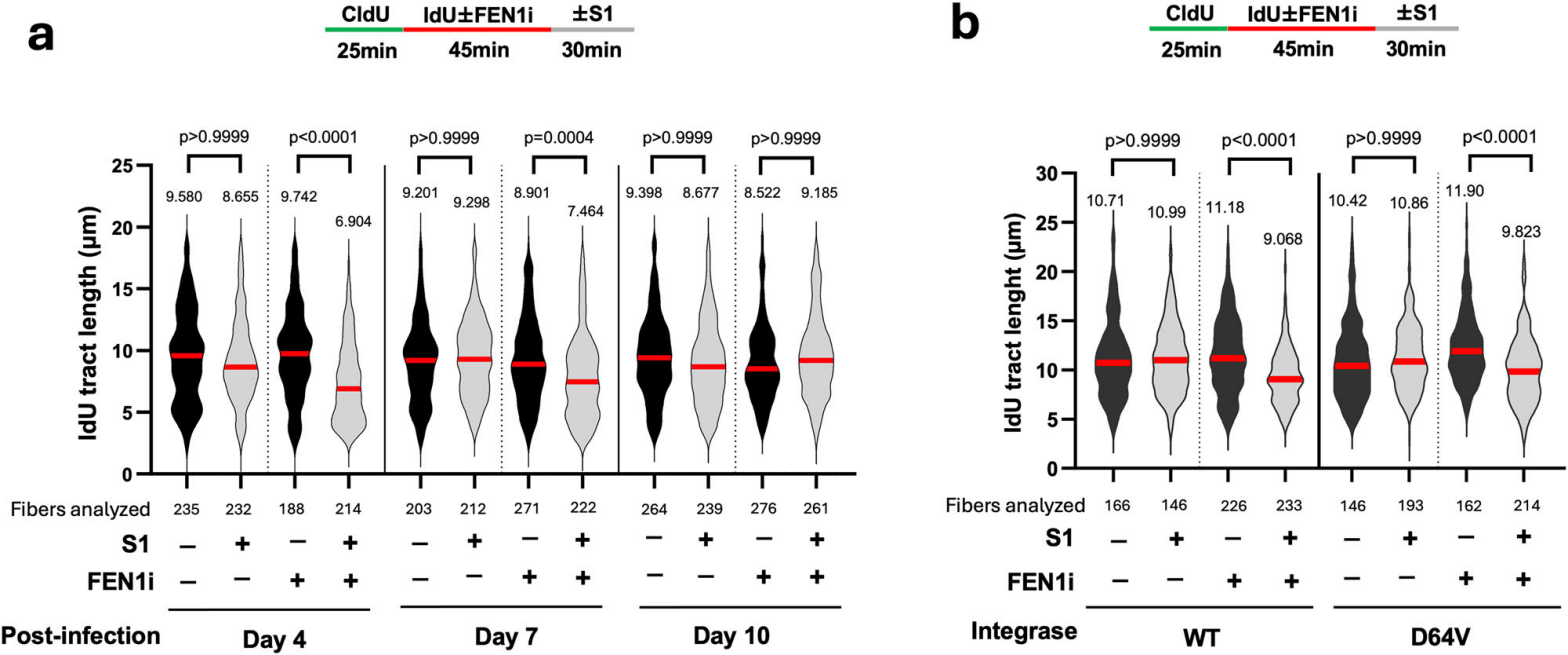
The effect of lentiviral infection on the S1 DNA fiber spreading assay is transient and does not require genome integration. **a** U2OS cells were infected with shLUC-encoding lentiviral particles and subjected to the S1 DNA fiber spreading analysis with and without FEN1 inhibitor four, seven, and ten days after infection. **b** U2OS cells were infected with the same amount of wild-type versus integration-deficient (D64V) lentiviral particles encoding YFP. The multiplicity of infection (MOI) was estimated to be between 3 and 30 based on p24-derived viral titers and the assumption that transducing units per ml are 100 to 1000-fold lower than physical units per ml. Four days after infection, cells were subjected to the S1 DNA fiber spreading assay with and without FEN1 inhibitor as described earlier. Median IdU tract lengths are shown above each experimental group and indicated by the red bars. The exact number of dual-colored DNA fibers analyzed is shown below each experimental condition. Three independent experiments were performed, which showed similar trends. *P* values are based on Kruskal–Wallis test with Dunnett’s multiple comparisons.

For lentiviral experiments, we typically treat cells with puromycin (the selection marker on the viral vectors) one day after infection for 2–3 days to ensure successful viral entry and stable expression of the transgenes before conducting downstream assays. Given that lentiviral vectors are stably integrated into the host genome, we asked if the virally-induced ability of the DNA spreading assay to detect strand-specific gaps is long-lasting. To test this, we infected U2OS cells with lentiviral  shLUC and performed the same DNA fiber spreading assay with and without FEN1 inhibitor at three different time points. We observed FEN1 inhibitor-induced ssDNA gaps on days 4 and 7, as has been shown in earlier experiments (Fig. 4a). However, the DNA gaps became undetectable on day 10. These data suggest that lentiviral infection triggers a transient cellular effect that enables the DNA fiber spreading assay to detect strand-specific gaps.

### Genome integration is not required for lentivirus to enable DNA fiber spreading assay to detect strand-specific DNA gaps

Given that genome integration is a key feature of lentivirus, we speculated that it may be important for the enabling effect of lentiviral infection on the DNA fiber assay. To test this, we generated YFP-encoding lentiviruses using the psPAX2 packaging plasmid expressing either wild-type integrase or its enzymatically inactive D64V mutant. We quantified the viral titers using the p24-based ELISA assay and infected U2OS cells with an equal number of viral particles. The multiplicity of infection (MOI) was estimated to be between 3 and 30 based on the assumption that 100–1000 physical viral particles contain one functional viral particle. Fiber quantification showed that FEN1 inhibitor-induced ssDNA gaps could be readily detected by the spreading assay following infection using both wild-type and integrase-deficient lentiviruses (Fig. 4b). Thus, our result suggests that genome integration of lentiviral DNA is not required for its ability to reveal strand-specific DNA gaps through DNA spreading.

### Lentiviral infection reduces cohesin level on nascent DNA in a transient manner

**Fig. 5:**
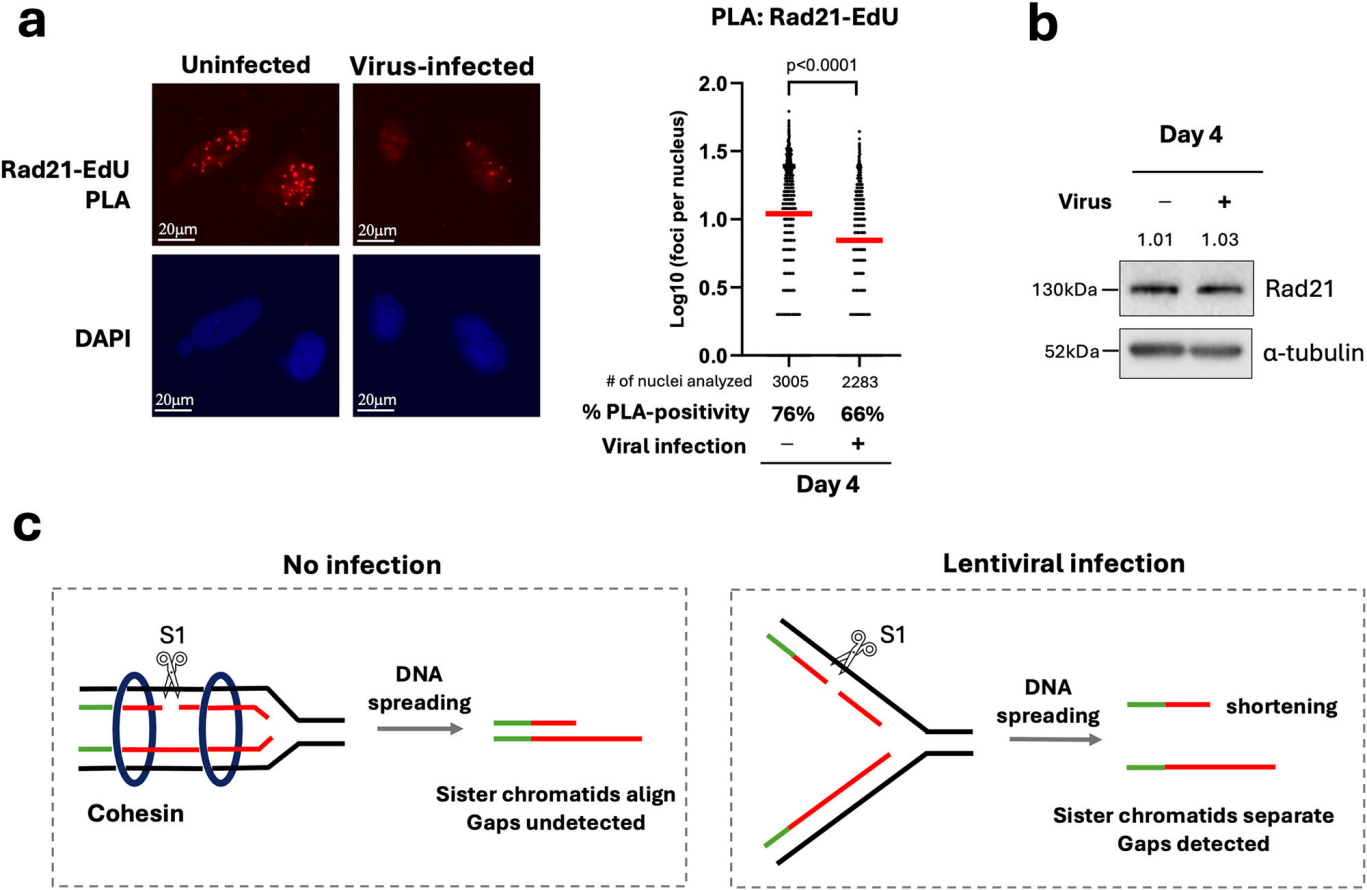
Lentiviral infection reduces cohesin level on nascent DNA. **a** U2OS cells were infected or not with shLUC-encoding lentiviral particles, and on day 4 post-infection, its nascent DNA was pulsed labeled for 20 min with 10 μM EdU. EdU was subsequently biotinylated through a Click reaction, and cells were subjected to proximity ligation assay (PLA) between Rad21 (core subunit of the cohesin complex) and Biotin. PLA foci per nucleus (based on DAPI) were quantified using Cell-Profiler and presented on a log_10_ scale. Median values are indicated as the red bars. The number of PLA-positive nuclei analyzed and the percentages of PLA-positive nuclei among all DAPI-positive nuclei are shown beneath each biological group. Four independent experiments were performed, which showed similar effects. *P* values were based on Mann–Whitney test. **b** Mock or shLUC lentivirus-infected U2OS cells were collected four days post-infection and subjected to Western blot for Rad21, with tubulin as the loading control. Rad21 bands were quantified by densitometry and expressed as relative values to tubulin. Five independent experiments were performed, which showed the same result. **c** Schematic illustrating the hypothetical mechanism by which lentiviral infection transiently enables the S1 DNA spreading assay to detect strand-specific ssDNA gaps, through a reduction of cohesin levels on nascent chromatin and the consequent separation of sister chromatids during DNA spreading.

Since the inability of the DNA fiber spreading assay to detect strand-specific DNA breaks is presumably due to sister chromatid cohesion^6^, we speculated that it may be affected by lentiviral infection. To test this, we performed proximity ligation assay (PLA) between the core subunit of the cohesin complex Rad21 and biotinylated EdU that was pulsed labeled into nascent DNA, using uninfected vs shLUC-infected U2OS cells four days post-transduction. PLA foci analysis revealed a significant reduction of Rad21 level on nascent DNA upon lentiviral infection (Fig. 5a). Notably, this was not due to the viral effect on the total expression level of Rad21 as Western blot analysis of whole cell lysates showed similar level of Rad21 between control and infected cells (Fig. 5b). Nor was the effect due to a decrease in EdU incorporation based on EdU-EdU PLA analysis (Supplementary Fig. 2a). Importantly, when analyzed ten days post-infection, at which point the viral effect on the S1 DNA spreading assay has subsided (Fig. 4a), Rad21-EdU PLA levels were similar between the control and virally infected cells (Supplementary Fig. 2b). Thus, our data suggest that lentiviral infection transiently reduces cohesin level on nascent DNA, allowing sister chromatid separation during DNA fiber spreading assay to reveal strand-specific ssDNA gaps (Fig. 5c).

## Discussion

As a well-established molecular technique, DNA fiber assay is instrumental to the study of replication fork dynamics under physiological and pathological conditions. Its value has been increasingly recognized in recent years due to the growing evidence that DNA replication deregulation strongly influences the efficacy of genotoxic anticancer treatments^24^. Of note, post-replicative ssDNA gaps have emerged as a common vulnerability and strong predictor of chemotherapy response of cancer, particularly in BRCA1/2-deficient tumors with impaired gap-filling capacity^7,8^. Post-replicative ssDNA gaps can arise on either the leading or lagging strand through distinct biological mechanisms. For example, defects in Okazaki fragment maturation, via either the canonical (FEN1/LIG1) or backup (PARP/XRCC1/LIG3) pathways, lead to ssDNA gaps on the lagging strand^11,25,26^, while overexpression or hyperactivation of the DNA-directed primase-polymerase PrimPol promotes aberrant repriming, likely on the leading strand, also causing ssDNA gaps^15,17,27^. The growing number of studies on ssDNA gaps reflects increasing interest in this area and has linked many factors to Okazaki fragment maturation and PrimPol-mediated repriming^7,8^. However, current understanding of the strand specificity of many gap regulators remains largely inferred, primarily due to the lack of simple and robust methods for strand-specific gap detection. Although the widely used S1-coupled DNA fiber spreading assay is reliable for detecting gaps, it can only do so when gaps are present on both daughter strands, as DNA spreading cannot disrupt tight sister chromatid cohesion^6^. This represents a major limitation in our ability to dissect detailed mechanisms of gap-regulation and fully exploit their therapeutic potential.

In this study, we described a previously unrecognized ability of lentiviral infection to render the standard S1 DNA spreading assay capable of detecting strand-specific gaps. We used FEN1 inhibitor to suppress Okazaki fragment maturation and specifically trigger lagging-strand DNA breaks^11^. Such gaps are invisible to DNA spreading but become readily detectable following lentiviral exposure of the host cells. We observed this in multiple cellular contexts and found no dependence on the viral vectors, the encoded sequences, or the ability of the viral DNA to integrate into the host genome. However, the effect was found to be transient and lasted for about seven days post-infection, at least based on the cell lines we examined. We found no evidence that virally infected cells are more prone to FEN1 inhibition, suggesting similar degrees of Okazaki fragment maturation defects and lagging-strand gap induction in the presence and absence of viral infection. Mechanistically, we detected reduced levels of cohesin, based on its core subunit RAD21, on nascent DNA upon lentiviral infection in a similarly transient manner. Notably, this occurred without detectable difference in RAD21 expression level, suggesting that reduced association and/or increased dissociation of cohesin from nascent DNA transiently allow the separation of sister chromatids during DNA spreading and the consequent detection of strand-specific gaps. This theory is further supported by the finding that nascent ssDNA gaps induced by PrimPol overexpression in otherwise unperturbed cells, theoretically on the leading strand, can only be detected by the DNA fiber spreading assay upon pre-exposure of the cells to lentivirus.

To our knowledge, the transient effect of lentivirus on cohesin/DNA interaction and sister chromatid cohesion has not been reported. Nonetheless, other types of human viruses have been reported to influence host genome structures by affecting cohesin interaction with DNA. For example, it was recently demonstrated that SARS-CoV-2 infection triggers selective depletion of cohesin from the intra-TAD (topological associating domain) regions of host chromatin. This was postulated to disrupt cohesin loop extrusion inside TADs to facilitate long-range chromatin interactions^28^. In another example, influenza A virus was shown to trigger local cohesin displacement from intragenic chromatin regions downstream of actively transcribed genes. This was attributed to virus-mediated inhibition of transcription termination and consequent RNA Pol II readthrough, which physically displaces cohesin^29^. The molecular mechanism underlying the transient lentivirus-mediated cohesin reduction from nascent DNA observed by us remains to be determined and may or may not be similar to those of other human viruses. However, such a transient and modest effect of lentivirus on cohesin association with chromatin is sufficient to explain how DNA spreading assay is rendered capable of detecting strand-specific gaps by prior lentiviral infection, which has important practical implications.

We propose that, to detect strand-specific DNA gaps, a simple modification can be made to the standard S1 DNA fiber assay by pre-infecting cells with lentiviral particles encoding a control transgene (e.g. YFP) or non-targeting shRNA (e.g. shLUC) four to seven days prior to the assay. We advise not to use freshly infected cells to allow the acute inflammatory response (24–72 h post-infection) to subside before performing the assay. Both purified and unpurified control viruses were effective in our assays. Based on our observations, infecting cells with control lentiviruses at 3-30 multiplicity of infection (MOI) had no detectable effect on their rates of DNA replication and proliferation, indicated by their similar doubling times and DNA fiber tract lengths compared to uninfected cells. Nor does the viral infection itself trigger detectable ssDNA gaps or replication stress on its own four days post-infection, at which point partial cohesion loss from chromatin is observed and DNA spreading assay is capable of detecting strand-specific gaps. Given the minimal effort needed to produce crude lentiviral particles and transduce host cells, incorporating this simple step in the DNA fiber assay is easily achievable and can potentially facilitate the discoveries of new mechanistic insights regarding strand-specific DNA alterations. In addition, since lentivirus-mediated gene delivery is frequently used in gain and loss-of-function studies, our findings suggest that, if assayed within 4–7 days post-infection, strand-specific DNA breaks can be detected by the standard DNA fiber spreading analysis. Nonetheless, it is conceivable that the experimental parameters used in our study, including viral titers and assay timelines may not be generalizable to all cellular contexts, and should be further optimized under different experimental conditions.

## Methods

### Cell lines

All cell lines were purchased from ATCC, authenticated, and tested for mycoplasma. U2OS, HeLa, and HEK293T cells were grown in high glucose DMEM containing 5% fetal bovine serum and 50 μg/mL gentamicin. MCF-7 and MDA-MB-231 cells were grown in RPMI 1640 containing 10% fetal bovine serum (FBS), 50 μg/mL gentamycin, 1 mM sodium pyruvate, 10 mM HEPES, and 4.5 g/L glucose.

### DNA constructs

Lentiviral vectors encoding YFP and shRNA targeting firefly luciferase (shLUC) were described previously^30^. Lentiviral vectors encoding shRNAs targeting human FEN1 (TTGCCGTCTTGTACCCTTAAG and GATGCCTCTATGAGCATTTAT) were purchased from Sigma. Lentiviral Myc-PrimPol plasmid was generated through PCR amplification of the Myc-PrimPol cDNA^19,31^ followed by insertion into the pFLRu-FH lentiviral vector as previously described^14,30,32^.

### Lentiviral production

To generate crude lentiviral particles, transfer DNAs encoding shRNAs or the yellow fluorescent protein (YFP) were co-transfected into HEK293T cells with the second-generation (pMD2.G together with psPAX2 or psPAX2-D64V) or third-generation packaging plasmids (pMDLg/pRRE, pCMV-G, pRSV-Rev). All constructs were purchased from Addgene except pCMV-G, which was provided by the Hope Center Viral Vectors Core at Washington University. For the second-generation packaging system, 10 μg transfer DNA were co-transfected with 2.5 μg pMD2.G and 8.5 μg pSPAX2 (WT or D64V) using 65 μL polyethylenimine (PEI) in 10 cm dishes. For the third-generation packaging system, 10 μg transfer DNA was co-transfected with 5.8 μg pMDLg/pRRE, 3.12 μg pCMV-G, and 2.52 μg pRSV-Rev using 65 μL PEI. Viral supernatants were collected two and four days after transfection, pooled, and stored as aliquots at −80 °C. Tittering of crude viral particles was performed using the p24-based QuickTiter Kit (Cell Biolabs, VPK-107-T) according to the manufacturer’s protocol. Purified lentivirus was generated by the Hope Center Viral Vectors Core at Washington University in St. Louis and tittered based on qPCR quantification of viral integration into the host cell genome.

### Viral infection of host cells

50–70 × 10^3^ cultured cells were plated in 6-well plates and infected on the following day with 1–2 ml of crude lentiviral supernatants for 4–6 h with the aid of 4 μg/mL polybrene. Puromycin was added at 1–2 μg/mL one day post-infection for 2–3 days till no viability could be detected in uninfected control cells. For purified virus, small amounts (0.25–2 μL) were diluted using fresh growth medium in the presence of 4 μg/mL polybrene to confer different multiplicities of infection (MOI). For vector control and Myc-PrimPol-expressing stable U2OS cells, they were generated by lentiviral infection and puromycin selection. Cells were grown in puromycin-free media for 2–3 weeks (to allow the initial lentiviral effects to fully subside) prior to the S1 DNA fiber analysis.

### S1 DNA fiber spreading assay

The S1 DNA fiber spreading assay was performed as previously described^33^. Briefly, 40–50 × 10^3^ cells were seeded in 12-well plates and pulsed labeled on the following day with 30 μM CldU (Sigma, C6891) for 20 min, then 250 μM IdU (Sigma, I7125) for 45 min with and without 10 μM FEN1 inhibitor LNT1 (Tocris Bioscience, 65–105). Cells were permeabilized for 3 min at RT with the detergent extraction buffer (100 mM NaCl, 20 mM HEPES (pH 7.4), 3 mM MgCl2, 300 mM sucrose, and 0.5% Triton X-100), washed with 1x S1 buffer (30 mM sodium acetate, 10 mM zinc acetate, 50 mM NaCl (pH 4.6), 5% glycerol), and incubated at 37 °C for 30 min in 1x S1 buffer with and without 20 U/ml S1 nuclease (Thermo Fisher, 18001016). Cells were subsequently scraped on ice in 0.5 ml PBS/0.1% BSA, pelleted at 4600 g for 5 min at 4 °C, and resuspended in PBS at 1500 cells/μL. 3 μL of resuspended nuclei were lysed on a positively charged glass slide (Thermo Fisher, 99-910-04) with 7 μL of lysis buffer (200 mM Tris-HCl (pH 7.5), 50 mM EDTA, and 0.5% SDS) for 4 min at RT. Slides were tilted to spread DNA, which was then air dried and fixed using freshly prepared methanol/acetic acid (3:1) for 5 min before storage at 4°C.

For immunodetection, DNA was denatured with 2.5 M HCl for 1 h at RT, washed in PBS, blocked with 5% BSA for 1 h, and co-incubated with mouse anti-BrdU (1:250, BD Biosciences, 347580) and rat anti-BrdU (1:100; Abcam, ab6326) for overnight at 4 °C or 1 h at 37 °C. Following washes with PBS/0.1% Tween 20, slides were incubated with anti-mouse Alexa Fluor 594 (1:500; Thermo Fisher, A21125) and anti-rat Alexa Fluor 488 (1:300; Thermo Fisher, A11006) for 1 h at RT, and mounted with ProLong Gold Antifade Reagent (Thermo Fisher, P36930). Fibers were imaged with a 60x oil objective on an epifluorescence microscope (Olympus IX70) using CellSens as the acquisition software. At least 150 dual-colored fibers were scored per biological condition using ImageJ. Each experiment was performed at least three independent times.

### Proximity ligation assay (PLA)

EdU-coupled PLA was performed as described previously^14^. Briefly, 15 × 10^3^ cells were seeded in 8-well Nunc Lab-Tek II-CC2 Chamber Slide (Sigma, S6815). On the following day, cells were pulsed for 20 min with 10 μM EdU, washed once in PBS, permeabilized for 5 min at RT in detergent extraction buffer (100 mM NaCl, 20 mM HEPES (pH 7.4), 3 mM MgCl2, 300 mM sucrose, and 0.5% Triton X-100), and fixed for 15 min at RT with 4% paraformaldehyde. After washing in PBS, cells were blocked for 1 h in PBS/5% BSA and subjected to Click-iT reaction for 30 min at RT in Click solution containing 20 µM biotin azide, 10 mM sodium ascorbate, and 2 mM CuSO4 in PBS, followed by overnight incubation with primary antibodies for Rad21 (1:200, Cell Signaling, #4321) and Biotin (1:1000, Jackson ImmunoResearch, 200-002-211 or 1:1000, Bethyl, #A150-109A) at 4 °C. On the following day, cells were subjected to the PLA procedure using different DuoLink products (Sigma) including probes (DUO92002 and DUO92004), in situ detection kit (DUO92008), and fluorescence wash buffers (DUO82049) according to the manufacturer’s protocols. Cells were mounted with ProLong Gold Antifade Reagent (Thermo Fisher, P36930) and imaged with a 40x objective on an epifluorescence microscope (Olympus IX70) using CellSens as the acquisition software. Images were processed using a custom pipeline built on the Cell-Profiler cell image analysis software to quantify the number of individual foci per nucleus. Over 3000 nuclei were analyzed per condition. Only the foci within the DAPI defined nucleus were quantified.

### Western blot

U2OS cells infected with lentiviral particles expressing shLUC or shFEN1 were harvested five days post-infection, lysed using RIPA buffer, quantified by Bradford assay, and analyzed by Western blot for FEN1 (1:1000, Cell Signaling, #83104) and alpha-tubulin (1:5000, Proteintech, 11224-1-AP). U2OS cells treated with 4 μg/ml polybrene alone or infected with shLUC in the presence of polybrene were collected after four days, lysed using RIPA buffer, and analyzed by Western blot for Rad21 (1:1000, Cell Signaling, #4321), pSer^784^-VCP (1:500, custom antibody^13^), pSer^4/8^-RPA32 (1:1000, Bethyl, #A300-245A), alpha-tubulin (1:5000, Proteintech, 11224-1-AP), and beta-actin (1:1000, Santa Cruz, sc-47778). Control or Myc-PrimPol-overexpressing U2OS cells were infected with shLUC or not and harvested four days later, lysed using RIPA buffer, and analyzed by Western blot for PrimPol (1:1000, Proteintech, 29824-1-AP) and beta-actin (1:1000, Santa Cruz, sc-47778).

### Statistics and reproducibility

Statistical significance for DNA fiber and PLA data was determined by Kruskal–Wallis test with Dunnett’s multiple comparisons or Mann–Whitney test (two-sided) depending on the number of experimental groups. Statistical significance for cell growth assay was based on a two-tailed Student's *t* test. Exact P values are shown in the graphs and considered statistically significant when they are less than 0.05. All results were confirmed by at least three independent experiments. Sample sizes for DNA fiber, PLA, and cell growth experiments are provided in the graphs and can be found in the Supplementary Data file.

### Reporting summary

Further information on research design is available in the Nature Portfolio Reporting Summary linked to this article.

## Supplementary information

Supplementary Information

Description of Additional Supplementary Files

Supplementary Data

Reporting Summary

## Data Availability

All data supporting the findings of this study are available within the paper and its Supplementary Information. Uncropped Western blot images are provided in the Supplementary Information. Numerical source data for all graphs are provided in the Supplementary Data.
