# Supplementary material for: Lentivirus enables the detection of strand-specific ssDNA gaps by the DNA fiber spreading assay
Source: Commun Biol. 2026 May 26;9:1115. doi: 10.1038/s42003-026-10367-w (PMC13482091; doi:10.1038/s42003-026-10367-w)
Supplement: Supplementary file 1 [file 42003_2026_10367_MOESM1_ESM.pdf]

Supplementary Figures

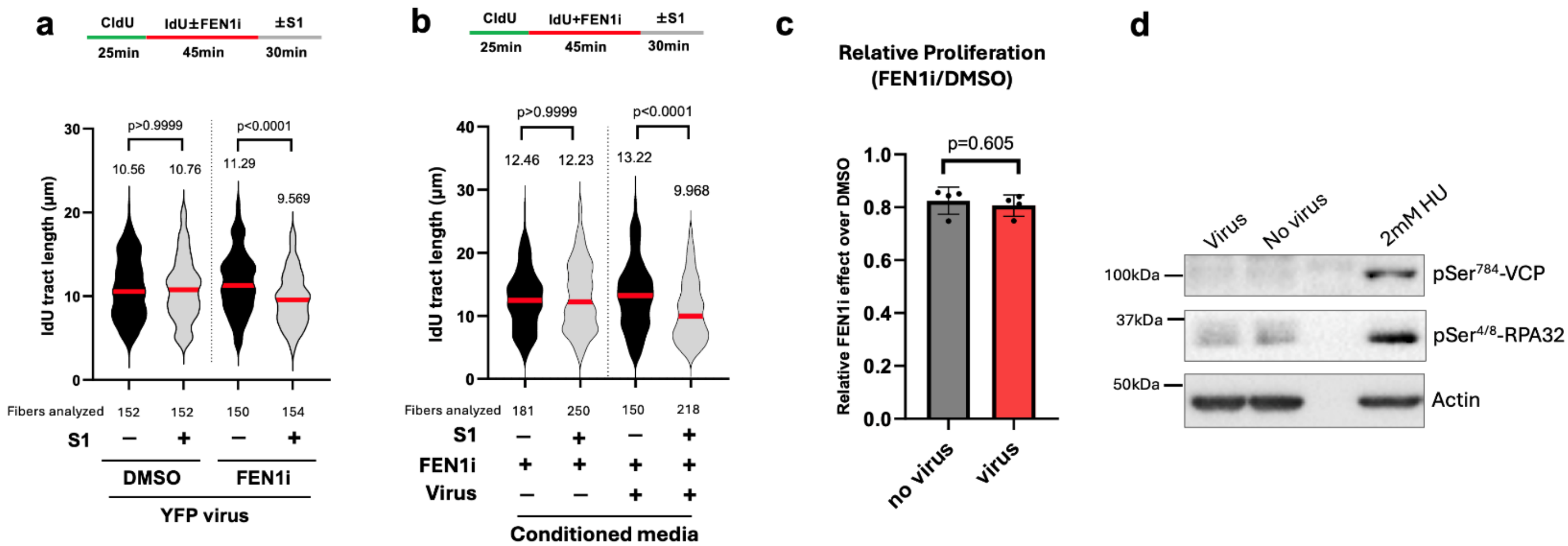

Supplementary Figure 1, related to Figure 2. (a) U2OS cells were infected with lentiviral particles encoding YFP in the pFLRu-FH vector, and subjected to the S1 DNA spreading assay with and without FEN1 inhibitor on day 4 post-infection. **(b)** U2OS cells were exposed to conditioned media lacking or containing shLUC lentivirus five days prior to the S1 DNA fiber spreading assay with FEN1 inhibitor. Median IdU tract lengths are shown above each experimental group and indicated by the red bars. The exact number of dual-colored DNA fibers analyzed is shown below each experimental condition. Three independent experiments were performed which showed similar trends. P values are based on Kruskal–Wallis test with Dunnett's multiple comparisons. **(c)** U2OS cells infected or not with shLUC lentivirus were seeded in the 96-well plate four days post-infection and treated with DMSO or 10 μM FEN1i LNT1 for three days. Viable cells were measured by Alamar blue and relative effects of FEN1i vs DMSO are shown as mean ± SD. P value is based on two-tailed student t-test. Two independent experiments were performed which showed no difference between virus and no virus groups. **(d)** U2OS cells infected or not with shLUC lentivirus were collected on day 4 and analyzed by Western blot for pSer^{784}-VCP and pSer$^{4/8}$-RPA32 with actin as loading control. Cells treated with 2 mM HU for 4 hours were used as the positive control. Two independent experiments were performed and showed the same trend.

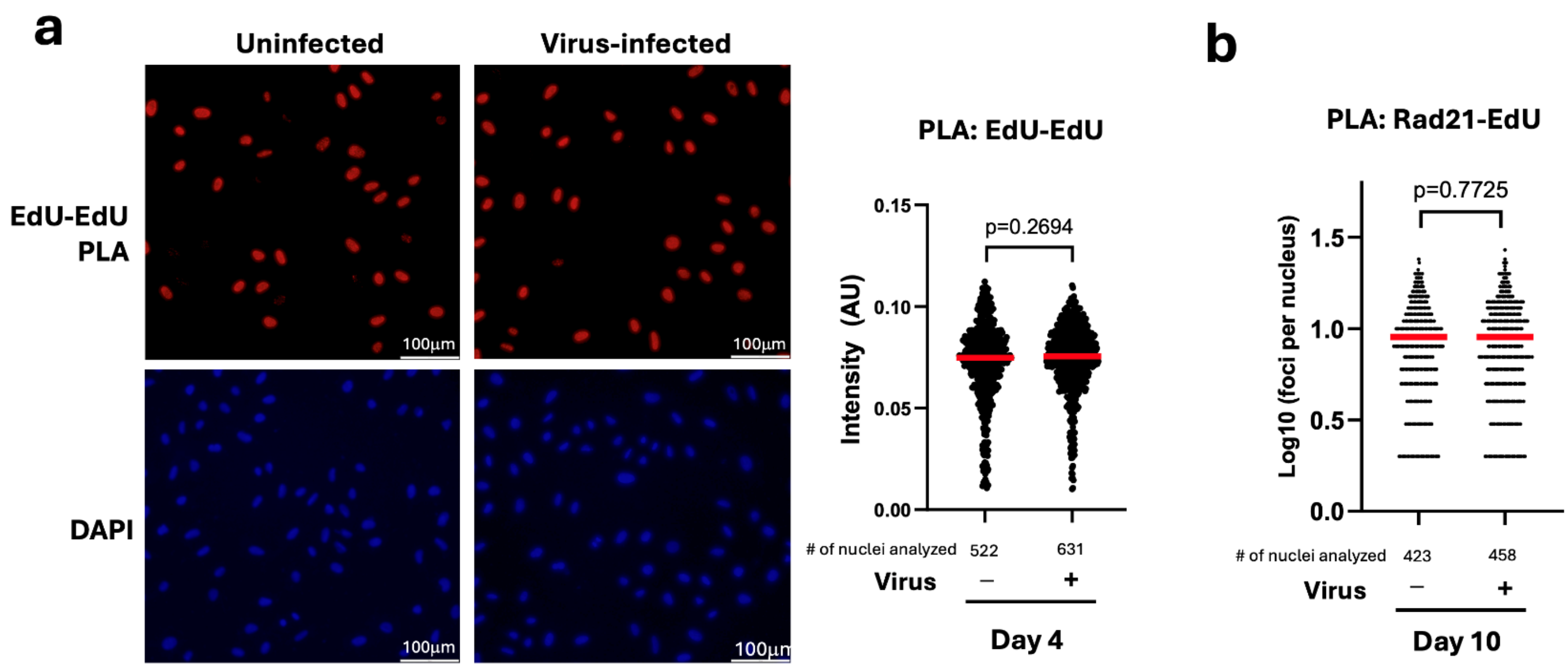

Supplementary Figure 2, related to Figure 5. (a) U2OS cells infected or not with shLUC lentivirus were pulsed labeled with 10 μM EdU for 20 min four days post-infection, and subjected to EdU-EdU PLA assay to assess EdU incorporation level. Representative images are shown on the left. Fluorescence intensities were quantified using Cell-Profiler, and positive values are graphed on the right with median values marked for each biological group. The number of analyzed nuclei are shown beneath the graph. P value is based on Mann–Whitney test. **(b)** U2OS cells infected or not with shLUC lentivirus were analyzed for Rad21-EdU PLA 10 days post-infection. The experimental procedure, as well as data presentation and analysis, are the same as in Fig. 5A.

Uncropped Western blot images

Figure 1c

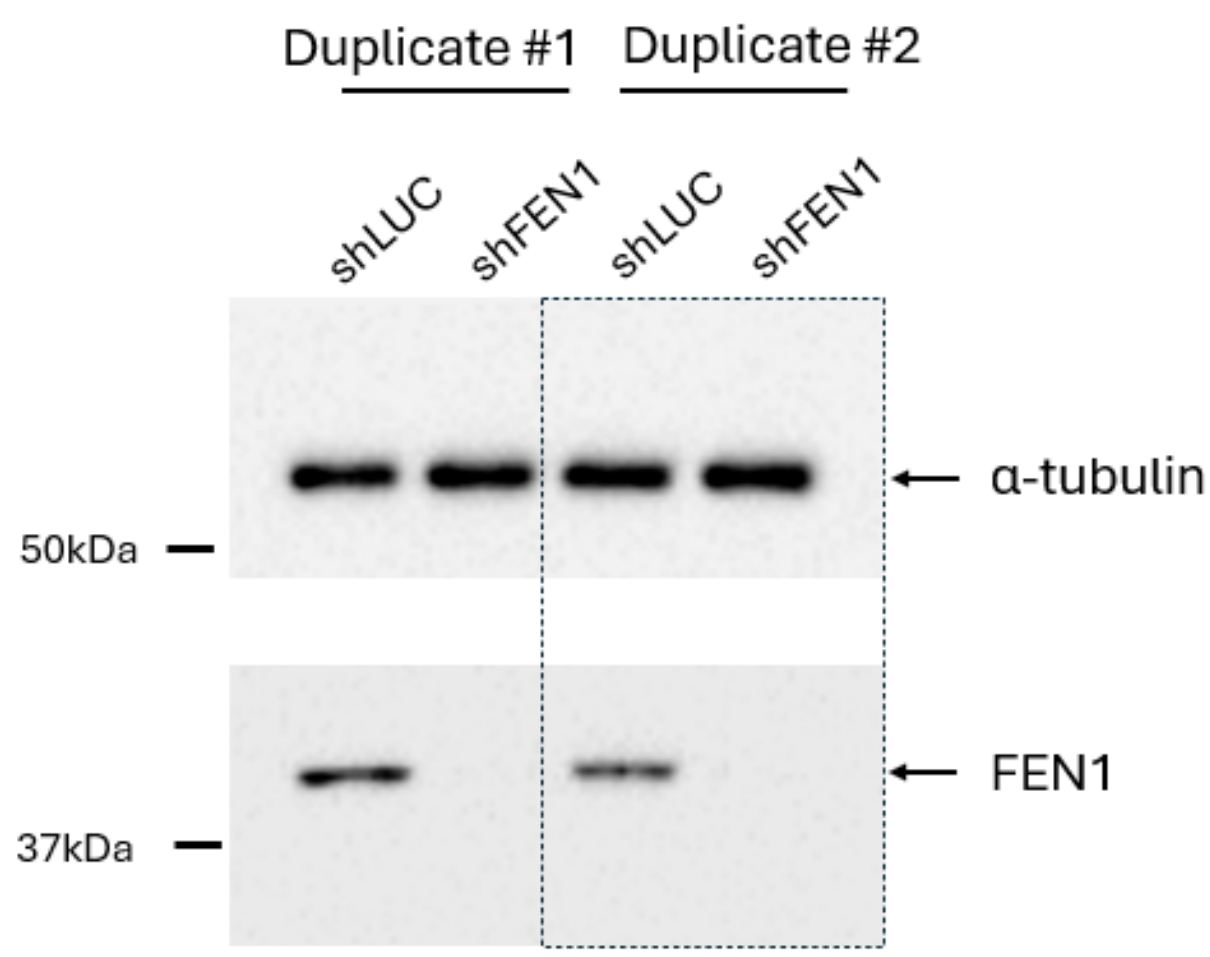

Boxed areas (duplicate samples #2) are shown in Figure 1c

Figure 2c

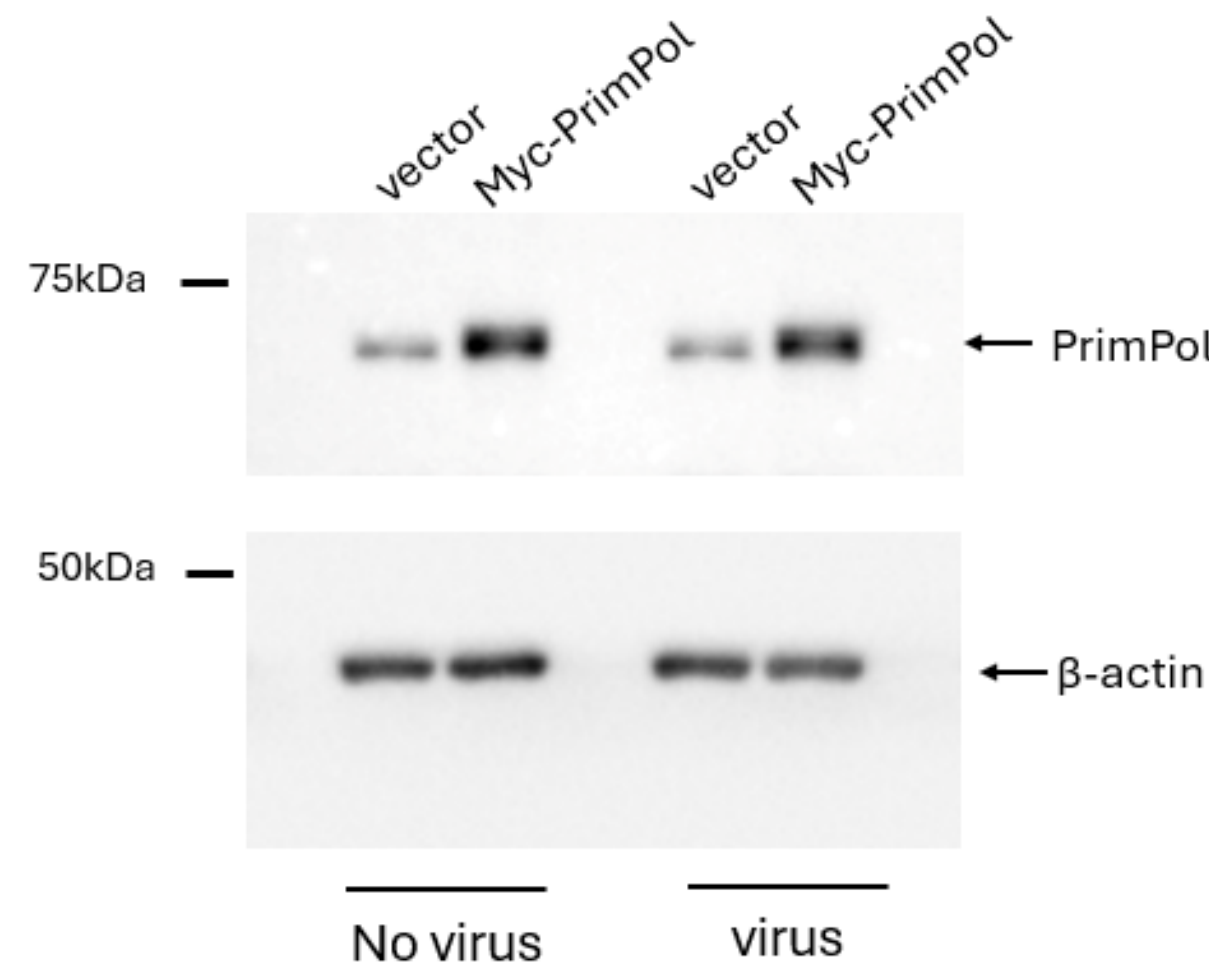

Figure 5b

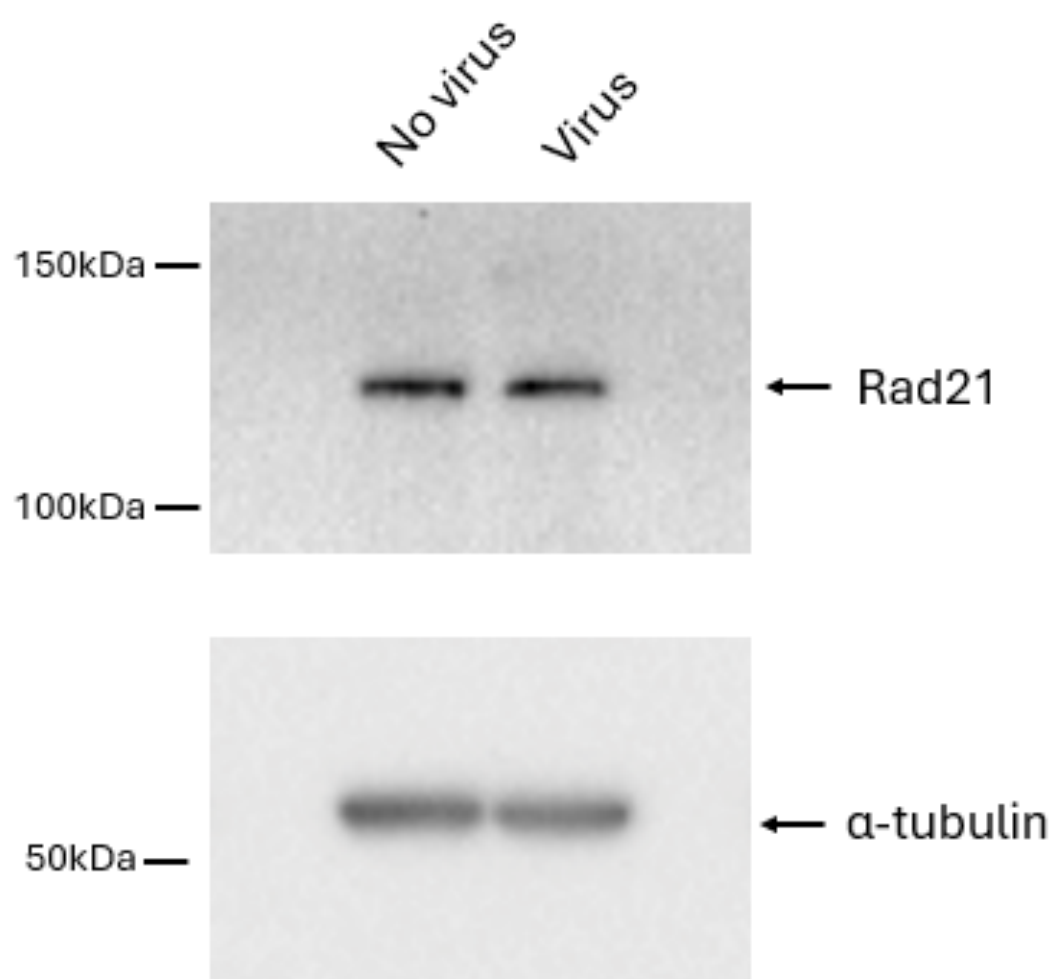

Supplementary Figure 1d

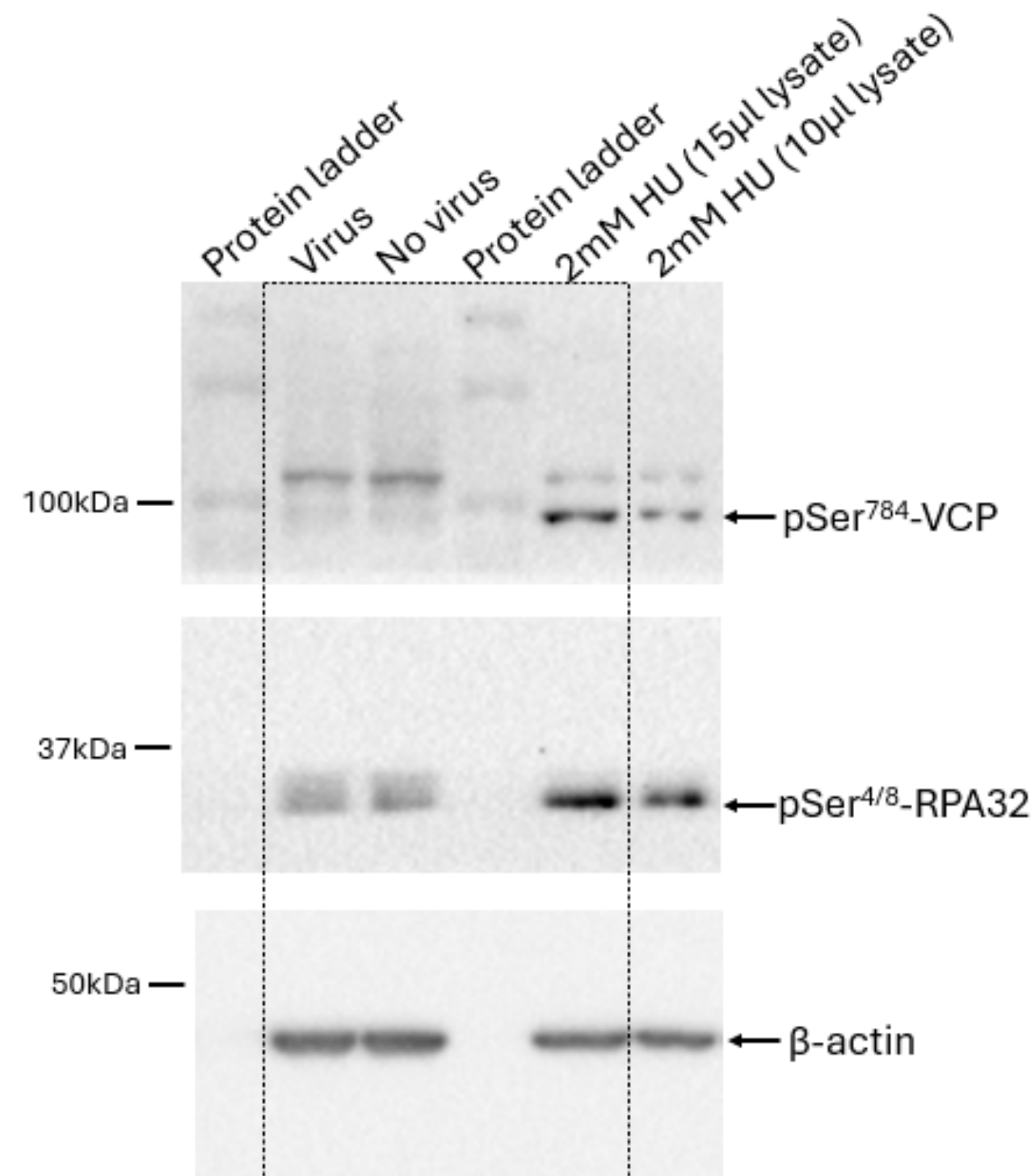

Boxed areas are shown in Supplementary Fig. 1d
