# Supplementary material for: Lentivirus enables the detection of strand-specific ssDNA gaps by the DNA fiber spreading assay
Source: Commun Biol. 2026 May 26;9:1115. doi: 10.1038/s42003-026-10367-w (PMC13482091; doi:10.1038/s42003-026-10367-w)
Supplement: Supplementary file 2 [file 42003_2026_10367_MOESM2_ESM.pdf]

Description of Additional Supplementary Files

File name- Supplementary Data

Description - Numerical source data for all 366 graphs are provided in the Supplementary Data.
