# Supplementary material for: Lentivirus enables the detection of strand-specific ssDNA gaps by the DNA fiber spreading assay
Source: Commun Biol. 2026 May 26;9:1115. doi: 10.1038/s42003-026-10367-w (PMC13482091; doi:10.1038/s42003-026-10367-w)
Supplement: Supplementary file 3 [file 42003_2026_10367_MOESM4_ESM.pdf]

nature portfolio

Corresponding author(s): Jieya Shao

Last updated by author(s): Mar 30, 2026

Reporting Summary

Nature Portfolio wishes to improve the reproducibility of the work that we publish. This form provides structure for consistency and transparency in reporting. For further information on Nature Portfolio policies, see our Editorial Policies and the Editorial Policy Checklist.

Statistics

For all statistical analyses, confirm that the following items are present in the figure legend, table legend, main text, or Methods section.

n/a	Confirmed	
☐	☒	The exact sample size (*n*) for each experimental group/condition, given as a discrete number and unit of measurement
☐	☒	A statement on whether measurements were taken from distinct samples or whether the same sample was measured repeatedly
☐	☒	The statistical test(s) used AND whether they are one- or two-sided<br>*Only common tests should be described solely by name; describe more complex techniques in the Methods section.*
☒	☐	A description of all covariates tested
☐	☒	A description of any assumptions or corrections, such as tests of normality and adjustment for multiple comparisons
☐	☒	A full description of the statistical parameters including central tendency (e.g. means) or other basic estimates (e.g. regression coefficient) AND variation (e.g. standard deviation) or associated estimates of uncertainty (e.g. confidence intervals)
☐	☒	For null hypothesis testing, the test statistic (e.g. *F*, *t*, *r*) with confidence intervals, effect sizes, degrees of freedom and *P* value noted<br>*Give P values as exact values whenever suitable.*
☒	☐	For Bayesian analysis, information on the choice of priors and Markov chain Monte Carlo settings
☒	☐	For hierarchical and complex designs, identification of the appropriate level for tests and full reporting of outcomes
☒	☐	Estimates of effect sizes (e.g. Cohen's *d*, Pearson's *r*), indicating how they were calculated

Our web collection on statistics for biologists contains articles on many of the points above.

Software and code

Policy information about availability of computer code

Data collection	Fluorescent images were collected using the standard CellSens software v4.3 on an epifluorescence microscope (Olympus IX70).<br>Western blot and colony formation images were acquired using the Image Lab software v6.1 installed on the Gel Doc XR imaging system (Bio-Rad)
Data analysis	DNA fibers were quantified by Image J v1.53t.<br>PLA images were analyzed by Cell-Profiler v4.2.8.<br>Statistical significance was determined by Prism v10.

For manuscripts utilizing custom algorithms or software that are central to the research but not yet described in published literature, software must be made available to editors and reviewers. We strongly encourage code deposition in a community repository (e.g. GitHub). See the Nature Portfolio guidelines for submitting code & software for further information.

Data

Policy information about availability of data

All manuscripts must include a data availability statement. This statement should provide the following information, where applicable:

- Accession codes, unique identifiers, or web links for publicly available datasets
- A description of any restrictions on data availability
- For clinical datasets or third party data, please ensure that the statement adheres to our policy

The authors declare that all data are available in the main manuscript and supplementary materials. Unprocessed Western blots will be provided as source data. Raw data for dot plots can be provided upon request.

Research involving human participants, their data, or biological material

Policy information about studies with human participants or human data. See also policy information about sex, gender (identity/presentation), and sexual orientation and race, ethnicity and racism.

Reporting on sex and gender	*Use the terms sex (biological attribute) and gender (shaped by social and cultural circumstances) carefully in order to avoid confusing both terms. Indicate if findings apply to only one sex or gender; describe whether sex and gender were considered in study design; whether sex and/or gender was determined based on self-reporting or assigned and methods used. Provide in the source data disaggregated sex and gender data, where this information has been collected, and if consent has been obtained for sharing of individual-level data; provide overall numbers in this Reporting Summary. Please state if this information has not been collected. Report sex- and gender-based analyses where performed, justify reasons for lack of sex- and gender-based analysis.*
Reporting on race, ethnicity, or other socially relevant groupings	*Please specify the socially constructed or socially relevant categorization variable(s) used in your manuscript and explain why they were used. Please note that such variables should not be used as proxies for other socially constructed/relevant variables (for example, race or ethnicity should not be used as a proxy for socioeconomic status). Provide clear definitions of the relevant terms used, how they were provided (by the participants/respondents, the researchers, or third parties), and the method(s) used to classify people into the different categories (e.g. self-report, census or administrative data, social media data, etc.) Please provide details about how you controlled for confounding variables in your analyses.*
Population characteristics	*Describe the covariate-relevant population characteristics of the human research participants (e.g. age, genotypic information, past and current diagnosis and treatment categories). If you filled out the behavioural & social sciences study design questions and have nothing to add here, write "See above."*
Recruitment	*Describe how participants were recruited. Outline any potential self-selection bias or other biases that may be present and how these are likely to impact results.*
Ethics oversight	*Identify the organization(s) that approved the study protocol.*

Note that full information on the approval of the study protocol must also be provided in the manuscript.

Field-specific reporting

Please select the one below that is the best fit for your research. If you are not sure, read the appropriate sections before making your selection.

☒ Life sciences ☐ Behavioural & social sciences ☐ Ecological, evolutionary & environmental sciences

For a reference copy of the document with all sections, see nature.com/documents/nr-reporting-summary-flat.pdf

Life sciences study design

All studies must disclose on these points even when the disclosure is negative.

Sample size	Sample sizes of our experiments were based on well-accepted standards in the field established by published studies (some of which are referenced below). They offered sufficient statistical power for us to draw reliable conclusions with regard to the specific biological questions. 1. For S1 DNA fiber assays, at least 150 fibers were analyzed in each sample and all data were confirmed by at least three independent experiments (Quinet et al, 2017, Methods Enzymol 591, 55; Kang et al., 2021 Nat Commun. 12, 5966; Peng et al., 2025 Mol Cell 85, 3373; Cong et al, 2021 Mol Cell 81, 3227; Quinet et al, 2020, Mol Cell 77, 461). 2. For proximity ligation assay, over 1000 nuclei were analyzed per condition and data were confirmed by three independent experiments (Higgs et al, 2018, Mol Cell 71, 25-41; Taglialatela et al, 2017, Mol Cell 68, 414-430).
Data exclusions	No data exclusions
Replication	All data in the paper were reproduced by at least three independent experiments.
Randomization	All images for DNA fiber and PLA assays were acquired in random fashion without preset orders of sample processing. In addition, efforts

Randomization	were taken to acquire images throughout the entire slides or wells to minimize positional effects.
Blinding	The authors were blinded to all manual analyses of DNA fiber data. No blinding was applied to PLA analysis as it was performed unbiasedly by software. No blinding was needed for Western blot as it is also immune to user bias.

Reporting for specific materials, systems and methods

We require information from authors about some types of materials, experimental systems and methods used in many studies. Here, indicate whether each material, system or method listed is relevant to your study. If you are not sure if a list item applies to your research, read the appropriate section before selecting a response.

Materials & experimental systems

n/a	Involved in the study
☐	☒ Antibodies
☐	☒ Eukaryotic cell lines
☒	☐ Palaeontology and archaeology
☒	☐ Animals and other organisms
☒	☐ Clinical data
☒	☐ Dual use research of concern
☒	☐ Plants

Methods

n/a	Involved in the study
☒	☐ ChIP-seq
☒	☐ Flow cytometry
☒	☐ MRI-based neuroimaging

Antibodies

Antibodies used	Primary antibodies used for Western blots are rabbit anti-FEN1 antibody (Cell Signaling, #83104), rabbit anti-Rad21 antibody (Cell Signaling, #4321), rabbit anti-PrimPol antibody (Proteintech, 29824-1-AP), rabbit pSer4/8-RPA32 antibody (Bethyl, #A300-245A), mouse pSer784-VCP antibody (custom antibody), mouse anti-actin antibody (Santa Cruz, sc-47778), and rabbit anti-tubulin antibody (Proteintech, #11224-1-AP). Primary antibodies used for DNA fiber assays are mouse anti-BrdU (Becton Dickinson, #37580) and rat anti-BrdU (Abcam, #ab6326). Primary antibodies used for PLA are mouse anti-Biotin (Jackson ImmunoResearch, #200-002-211), rabbit anti-Biotin (Bethyl, #A150-109A), and rabbit anti-Rad21 (Cell Signaling, #4321). Secondary antibodies used for Western blots are horseradish peroxidase-conjugated anti-rabbit (CST, #7074) and anti-mouse (CST, #7076). Secondary antibodies for immunofluorescence are Alexa Fluor 594-conjugated goat anti-mouse IgG (H+L) (ThermoFisher, #A21125) and Alexa Fluor 488 -conjugated goat anti-rat IgG (H+L) (ThermoFisher, #A11006).
Validation	For anti-FEN1 antibody, its specificity was validated by FEN1 KD in this study (Fig.1B). For anti-PrimPol antibody, its specificity was validated by PrimPol KD and the result is provided on the company's website. For anti-Rad21 antibody, its specificity was validated by Rad51 deletion by Dong et al., Nature Genetics, 2024; 56(8), 1654–1664. For pSer784-VCP antibody, its specificity was validated by Zhu et al., Cell Rep., 2020; 31(10):107745. For pSer4/8-RPA32 antibody, its specificity has been validated both by the manufacturer and numerous published papers such as Zuazua-Villar et al., NAR, 2015; 20:9776–9787.<br><br>For anti-BrdU and anti-Biotin antibodies, their specificities for DNA fiber and PLA assays have been well-established by numerous published studies.

Eukaryotic cell lines

Policy information about cell lines and Sex and Gender in Research

Cell line source(s)	U2OS, HeLa, MCF-7, MDA-MB-231, and HEK293T cells were purchased from ATCC.
Authentication	All cell lines show their distinctive growth and morphological behaviors as expected based on information from ATCC and published studies. As such, no authentication was performed.
Mycoplasma contamination	All cell lines were tested negative for mycoplasma contamination.
Commonly misidentified lines (See ICLAC register)	No commonly misidentified cell lines were used in the study.

Plants

Seed stocks	*Report on the source of all seed stocks or other plant material used. If applicable, state the seed stock centre and catalogue number. If plant specimens were collected from the field, describe the collection location, date and sampling procedures.*
Novel plant genotypes	*Describe the methods by which all novel plant genotypes were produced. This includes those generated by transgenic approaches, gene editing, chemical/radiation-based mutagenesis and hybridization. For transgenic lines, describe the transformation method, the number of independent lines analyzed and the generation upon which experiments were performed. For gene-edited lines, describe the editor used, the endogenous sequence targeted for editing, the targeting guide RNA sequence (if applicable) and how the editor was applied.*
Authentication	*Describe any authentication procedures for each seed stock used or novel genotype generated. Describe any experiments used to assess the effect of a mutation and, where applicable, how potential secondary effects (e.g. second site T-DNA insertions, mosiacism, off-target gene editing) were examined.*
